# The effects of COVID-19 pandemic countermeasures on patients receiving botulinum toxin therapy and on their caregivers: a study from an Italian cohort

**DOI:** 10.1007/s10072-021-05282-3

**Published:** 2021-05-06

**Authors:** Domiziano Tarantino, Rossana Gnasso, Federico Migliore, Irene Iommazzo, Felice Sirico, Bruno Corrado

**Affiliations:** Department of Public Health, University Federico II of Naples, Naples, Italy

**Keywords:** Cerebral palsy, Botulinum Toxin, SARS-CoV-2, COVID-19, Coronavirus, Caregiver burden

## Abstract

COVID-19 outbreak had a huge worldwide impact, and several countermeasures to contain its spread have been adopted, such as the interruption of nonurgent outpatient clinics. We wanted to describe the effects of the national lockdown on the well-being of a cohort of Italian patients with cerebral palsy (CP) receiving botulinum toxin (BT) therapy and of their caregivers. Twenty-five patients receiving BT therapy were surveyed using the structuralized questionnaire by Dressler and Adib Saberi, while the caregivers were assessed using the Caregiver Burden Scale. The lockdown delayed BT therapy by 9 ± 2.8 months. Around 44% of the selected patients noticed increased muscle cramps, 24% increased muscle pain, and 32% both of them. Due to the lockdown, the patient’s quality of life was reduced by 68.4 ± 21.1%. After the lockdown, 100% of patients perceived BT therapy as more important than before. Around 76% of the patients perceived the lockdown as inadequate and felt that their rights were not respected. The overall score of the Caregiver Burden Scale, as regarded before the lockdown, was 29.12 ± 11.63, while the overall score as regarded after the lockdown was 37.44 ± 14.85. The overall score increased, from before the lockdown to after the lockdown, for 92% of caregivers. The BT outpatient clinic’s interruption was seen to significantly worsen the psychophysical condition of subjects with CP and the care burden of their caregivers, exposing them to greater stress than before. Therefore, any kind of BT treatment suspension or delay should be avoided.

## Introduction

The severe acute respiratory syndrome coronavirus 2 (SARS-CoV-2) and its related disease (coronavirus disease 2019, also known as COVID-19) outbreak had a huge impact during 2020. Italy was the first country to be affected in Europe and among the most affected in the world after China, with a number of reported cases being the highest in Europe during the first outbreak of the pandemic [1, 2]. Consequently, several countermeasures to contain the COVID-19 spread were adopted, such as a general lockdown and the interruption of public hospitals’ outpatient clinics [1]. This interruption put under pressure most healthcare sectors, including the physical and rehabilitation medicine (PRM) ones, and negatively influenced the rehabilitation process of patients with severe neuromotor impairments, such as subjects affected by cerebral palsy (CP) receiving botulin toxin (BT) therapy together with physiotherapy.

Patients with CP usually develop muscle hypertonia and joint contractures, which cause moderate to severe disability degrees. As a consequence, the daily care of these patients from their caregivers can be very difficult. The best way to increase and maintain muscle elasticity, and to prevent contractures, is undergoing physical therapy. Other kinds of therapy can be added to physical therapy in order to improve the outcomes, such as oral medications, splinting and casting, BT therapy, intrathecal baclofen, extracorporeal shockwave therapy, and orthopedic surgery [3–7].

BT therapy was proven to be a safe and effective therapy, and it is widely used for the treatment of local spasticity in patients with CP. However, BT therapy has short-term effects on muscle hypertonia, so it must be frequently repeated. For this reason, we hypothesized that the interruption of BT therapy due to COVID-19 countermeasures could have a significant impact on the physical condition of patients with CP, also causing many troubles to their caregivers.

Therefore, in this study, we wanted to describe how the countermeasures for COVID-19 influenced the well-being of a cohort of Italian patients with CP and their caregivers.

### SARS-Cov-2 countermeasures

In order to limit the COVID-19 spread, the president of the Italian Republic promulgated a decree on 23 February 2020, pointing out the need to draw up “urgent measures on the containment and management of the epidemiological emergency due to COVID-19” [8]. Basing on this national decree, the Campania region (with Naples being its county seat) that was one of the first regions in Italy to adopt all the necessary countermeasures against COVID-19 promulgated a regional regulation on 5 March 2020 that imposed the immediate suspension of all the deferrable outpatient activities provided by all kind of public and private hospitals until 18 March 2020 [9].

The University Hospital Federico II of Naples, where our BT clinic is based and this survey was carried out, followed the regional regulation of 5 March 2020 and then extended the interruption of nonurgent outpatient activities until 3 May 2020 [9].

From 3 May to September 2020, despite the restart of some outpatient clinics, our BT clinic was not operative since the caregivers of patients with CP did not agree to resume BT therapy, having fear of a higher risk of infection in the hospital setting.

## Methods

### Design

This study is a survey on the lockdown’s effects due to the SARS-CoV-2 pandemic on both patients receiving BT therapy for CP and their caregivers. The survey is based on a face-to-face interview.

Two different questionnaires were used: one was extracted from the article by Dressler and Adib Saberi [10] (Table 1), while the other is the Caregiver Burden Scale [11, 12].

**Table 1:**
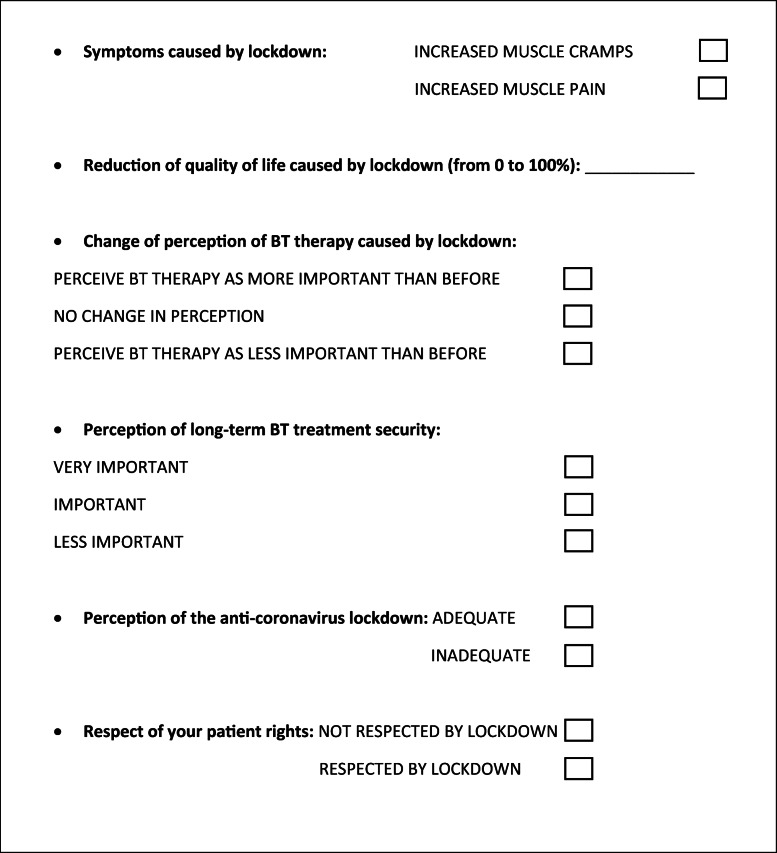
Structuralized questionnaire to survey the effects of the anticoronavirus lockdown on patients receiving BT therapy by Dressler and Adib Saberi [10], modified by the lead author Dr. Domiziano Tarantino

The structuralized questionnaire by Dressler and Adib Saberi [10] investigates the effects of the anticoronavirus lockdown on patients receiving BT therapy, and it is composed of 6 items that analyze different aspects of patient’s well-being (symptoms, quality of life, change of perception of BT therapy, perception of long-term BT therapy security, perception of anticorona lockdown, and respect of patient’s rights).

The Caregiver Burden Scale questionnaire [11, 12] is composed of 22 questions grouped into five groups, covering important areas for caregivers, such as health, mental well-being, personal relationships, physical overload, social support, finances, and home environment. A total score from 0 to 20 means little or no burden, from 21 to 40 means mild to moderate burden, from 41 to 60 means moderate to severe burden, and from 61 to 88 means severe burden. For the purpose of our study, in order to evaluate how the overall caregivers’ quality of life was affected by the lockdown, they were asked to fill the questionnaire two times: one considering the period before the lockdown and the other after the lockdown.

The questionnaires were filled in when our BT outpatient clinic restarted its activity that was in September 2020. The last questionnaire was completed in December 2020.

This study was carried out following the guidelines given by the local ethics committee. All information from patients and their caregivers were treated anonymously, and all data were saved on a laptop which access password was given just to the authors involved in the study.

### Treatment institution

The study was performed at the Rehabilitation Unit, Department of Public Health, University of Naples Federico II, Naples, Italy. The BT outpatient clinic was founded in 2014. Four resident doctors (D.T., R.G., F.M., I.I.) and two professors of medicine (F.S., B.C.) were involved in the study. In 2019, 7525 IU (international unit) of BT were administered, while in 2020, due to the lockdown and the subsequent closure of the BT outpatient clinic, only 4480 IU of BT were administered.

### Patients

Patients were consecutively enrolled after the restart of the activities of the BT outpatient clinic. A total of 25 patients were recruited for this study. The inclusion criteria were the following: (1) BT therapy for CP; (2) patients with mild to moderate cognitive impairment; (3) age range 11–31 years; (4) BT therapy from at least 1 year; (5) BT therapy interrupted due to the lockdown; and (6) ability from both patients and caregivers to understand and to react adequately to questionnaires. The participation of patients and caregivers in this study was entirely voluntary, and none of the invited patients declined their participation.

### BT therapy

BT therapy was performed with onabotulinumtoxinA (100 IU in 2 ml 0.9% NaCl/H_2_O, 5 IU per 0.1 ml). BT therapy was available to all patients at no cost because patients with CP have an exemption from payment. Interinjection intervals were usually settled between 4 and 6 months, and the BT doses were decided by the entire group of the authors after a clinical examination of the patient.

As shown in Table 2, The BT total doses administered for patients in 2020 were 180 ± 88.32 IU (min. 100 IU, max. 350 IU). The patients treated in 2020 received BT therapy for 3.4 ± 1.7 years. Before the lockdown, BT therapy was applied to the patients treated in 2020 at intervals of 5 ± 1.8 months.

**Table 2:** Patient demographics and BT therapy data

Total number of patients [*n*]	25
Male patients [*n*]	16
Female patients [*n*]	9
Patient age (mean ± standard deviation) [years]	19.8 ± 7.9
Indication for BT therapy	Complications of CP
BT drug	OnabotulinumtoxinA
BT total equivalent dose in 2020 (mean ± standard deviation) [IU]	180 ± 88.32
Treatment duration (mean ± standard deviation) [years]	3.4 ± 1.7
Interinjection interval before lockdown (mean ± standard deviation) [months]	5 ± 1.8

## Results

### Patients

As shown in Table 2, a total of 25 patients (age 19.8 ± 7.9, 16 males, 9 females) were included in this study. All patients suffered from complications of CP (such as hemiplegia, diplegia, tetraplegia, and cognitive impairment).

### Lockdown effects

Table 3 shows the effects of the lockdown on the patients receiving BT therapy. As a result of the lockdown, the interinjection intervals increased from 5 ± 1.8 to 14 ± 3.3 months, delaying BT therapy by 9 ± 2.8 months. The minimum delay was (by definition) 10 months; the maximum delay was 19 months.

**Table 3:** Effects of the BT outpatient clinic lockdown on patients and their well-being according to the questionnaire by Dressler and Adib Saberi [10]

Delay of retreatment (mean ± standard deviation) [months]	9 ± 2.8
Symptoms caused by lockdown	
• Increased muscle cramps [% of patients] • Increased muscle pain [% of patients] • Increased both of them [% of patients]	44 24 32
Reduction of quality of life caused by lockdown (mean ± standard deviation) [%]	68.4 ± 21.1
Change of perception of BT therapy caused by lockdown	
• BT therapy is more important than before [% of patients] • No change [% of patients] • BT therapy is less important than before [% of patients]	100 0 0
Perception of long-term BT treatment security	
• Very important [% of patients] • Important [% of patients] • Less important [% of patients]	76 24 0
Perception of lockdown	
• Inadequate [% of patients] • Adequate [% of patients]	76 24
Respect of patient’s right	
• Not respected [% of patients] • Respected [% of patients]	76 24

Regarding the structuralized questionnaire created by Dressler and Adib Saberi [10], 44% of the patients noticed increased muscle cramps, 24% increased muscle pain, and 32% both of them. Due to the lockdown, the patient’s quality of life was reduced by 68.4 ± 21.1%. After the lockdown, all patients (100%) perceived BT therapy as more important than before. Around 76% of patients perceived long-term security of BT therapy availability as very important, 24% as important, and none as less important. Around 76% of the patients perceived the lockdown as inadequate and felt that their patient rights were not respected, while 24% of patients perceived the lockdown as adequate and felt that their patient rights were respected.

Table 4 shows the effects of the lockdown on patients’ caregivers according to the Caregiver Burden Scale. The overall score of the Caregiver Burden Scale questionnaire [11, 12], as regarded before the lockdown, was 29.12 ± 11.63, with a minimum score of 10 and a maximum of 49. The overall score as regarded after the lockdown was 37.44 ± 14.85, with a minimum score of 13 and a maximum of 65. From before the lockdown to after the lockdown, the overall score increased in the opinion of 92% of caregivers (23/25), while in the opinion of the remaining 8% (2/25), the score remained the same. The scores related to some specific questions increased, from before to after the lockdown, more than others. The score that increased the most (68% of caregivers) was the one related to question number 7 that states “Are you afraid about what the future holds for your relative?” The other scores that increased in more than 50% of caregivers are reported in Table 4.

**Table 4:** Effects of the lockdown on patients’ caregivers according to the Caregiver Burden Scale [11, 12]

Overall score before the lockdown (mean ± standard deviation) • Minimum score • Maximum score	29.12 ± 11.63 10 49
Overall score after the lockdown (mean ± standard deviation) • Minimum score • Maximum score	37.44 ± 14.85 13 65
Score change from before to after the lockdown [% of caregivers]	
• Increased • No change • Decreased	92 8 0
Q.1: “Do you feel that your relative asks for more help than he or she needs”? [% of caregivers]	60%
Q.2: “Do you feel that because of the time you spend with your relative, you do not have enough time for yourself?” [% of caregivers]	60%
Q.7: “Are you afraid about what the future holds for your relative”? [% of caregivers]	68%
Q.9: “Do you feel strained when you are around your relative”? [% of caregivers]	52%
Q.22: “Overall, how burdened do you feel in caring for your relative”? [% of caregivers]	60%

*Q*, question

## Discussion

The COVID-19 pandemic caused the interruption of public hospitals’ outpatient clinics in order to contain its spread, especially among the most fragile patients. The COVID-19 pandemic had a negative effect on the care of patients with neurologic conditions since it is well known that all chronic neurological conditions require a regular and accurate follow-up [13, 14]. PRM clinical activities were heavily affected, negatively influencing the rehabilitation process of subjects affected by neuromotor disorders, such as patients with CP receiving BT therapy.

In this survey, we pointed out that the lockdown increased the interinjection intervals from 5 ± 1.8 to 14 ± 3.3 months, delaying BT therapy by 9 ± 2.8 months; the maximum delay registered was 19 months, so the biological effects of BT were lost due to this important delay in its administration. Consequently, patients’ quality of life during this period was reduced by 68.4 ± 21.1%. In 100% of patients, the lockdown confirmed the perception of the importance of BT treatment. More than half of the patients (76%) felt the long-term BT treatment security as very important for their health condition and reported that their patient rights were not respected during the lockdown. Therefore, the pandemic and the subsequent interruption of BT therapy for patients with CP caused a worsening of their psychophysical conditions, with a marked increase in muscle stiffness and perceived pain.

The management by caregivers has thus become remarkably more difficult, as shown by the results of the Caregiver Burden Scale that assessed the extent of the burden on caregivers in the management of these complex and very demanding patients. Before the lockdown, the overall score of the Caregiver Burden Scale was 29.12 ± 11.63, while after the lockdown, it increased to 37.44 ± 14.85. The overall score increased, from before the lockdown to after the lockdown, for 92% of caregivers, and only for the 8%, it remained the same. It was clear that the suspension of BT therapy worsened both spasticity and pain of these patients, making their management more difficult than the past and also making caregivers’ lives more stressful. In 5 of the 22 questions of the Caregiver Burden Scale, the score increased considerably from before to after the lockdown.

More than the half of caregivers reported a feeling of tension and discomfort, and three questions that impacted the emotional sphere of caregivers highlighted how, for more than 60% of them, the lockdown’s limitations lead to an increased feeling of frustration and exhaustion in the management of the relatives with CP. Furthermore, 68% of caregivers were afraid of what the future could hold for their relatives, more than they had before the outbreak of the pandemic.

It was clear that a global phenomenon with this impact highlighted the disadvantage of patients with chronic diseases, such as subjects with CP for whom continuous social and health assistance are needed. The inability to guarantee these kinds of assistance during the lockdown leads to an increased fear for caregivers who were afraid of the negative effects that another similar situation could have on their relatives in the future.

Our results can be somehow compared with those studies conducted by other rehabilitation departments in Italy and abroad that assessed the impact of the COVID-19 pandemic on patients treated with BT for spasticity.

Samadzadeh et al. [15] carried out an observational study on the impact of SARS-CoV-2 pandemic lockdown on a BT outpatient clinic, analyzing the effect of SARS-CoV-2-induced reinjection delay on outcomes in neurological patients with an appointment between 20 April and 18 May during the partial lockdown in Germany. Of the 94 participating patients, 48 reported a delay and 44 a worsening during the delay. Delays ranged from 1 to 63 days; the mean delay was 23 days, and the mean worsening was 26% in comparison to the previous BT treatment. A remarkable correlation was shown between the duration of the delay and the patient’s rating of worsening: 1 day of delay caused 1% worsening and a delay of 3 to 4 weeks leads to a worsening of about 25%. These data highlighted that even a small BT interruption of a few weeks can lead to an important worsening of symptoms and that only a regular treatment up to at least 1 year would be needed to reach the same level of BT treatment benefits as that before the COVID-19 outbreak. For this reason, the authors stated that BT therapy should not be interrupted during the second national lockdown in order to avoid further dramatic consequences for patients treated with BT therapy [15].

Santamato et al. [16] conducted, from March to May 2020, a phone-based survey about patients with spasticity after stroke and traumatic brain injury in treatment with BT, submitting to 151 subjects an ad hoc questionnaire named CORTOX (CORonavirus TOXin survey) to investigate how much their health perception and well-being were influenced by the discontinuation of their BT treatment due to the lockdown. Around 72.2% of participants experienced a worsening in perceived spasticity, and 70.9% had a negative impact on quality of life. Moreover, more than half of patients (53%) reported a loss of their independence as a result of COVID-19-related problems and the interruption of rehabilitation services [16].

Ranza et al. [17] re-organized their work with the support of digital technology, monitoring patients with spasticity via phone calls and telemedicine. Phone calls were found to be the more useful system since several patients were contacted and triaged with success, while telemedicine had different limitations, such as the inability to perform a full physical examination and to adequately evaluate spasticity, contracture, and muscle stiffness. Another issue related to telemedicine was the difficulty in using digital devices by most spastic subjects. As in our survey, the authors suggested that the neurorehabilitation cannot be suspended since the lockdown increased the interinjection intervals for most patients; worsened their spasticity; had a negative impact on them, requiring a greater need for care; and also decreased their quality of life and limited their nursing assistance. In their conclusion, telemedicine was not an efficacy alternative to in-person assessment, but it can support the identification of subjects who could be more affected by the suspension of BT treatment [17].

Erro et al. [18] carried out a case-control study about the effects of the BT outpatient clinic interruption on spastic patients and their health-related quality of life (HRQoL). Soon after the beginning of lockdown’s countermeasures in Italy, 137 patients (94 cases and 43 controls) were asked to rate their perceived worsening from the time of their last treatment using a visual analog scale and a standardized, two-component tool to evaluate their health status. They found that the case group reported a mean delay of therapy of about 73 days and a remarkable worsening of their condition than the control group (5.16 ± 3.09 vs 1.83 ± 3.34). Anyway, although the pandemic had important clinical consequences on neurological patients who suspended BT therapy, this evidence was not mirrored by a negative effect on HRQoL [18].

One limitation of our study is the relatively small sample and the lack of a control group, but it should be emphasized that, to our knowledge, this is the first survey that assessed the impact of COVID-19 countermeasures not just on a relatively young population (i.e., <31 years) receiving BT therapy but also on their caregivers, focusing on how much lockdown caused for them significant economic, social, physical, and psychological management difficulties.

In conclusion, the lockdown confirmed the considerable burden caused by CP and its related neuromotor impairment on patients and their management by caregivers. The results of our study highlighted how the BT outpatient clinic’s interruption in 2020 worsened the psychophysical condition of subjects with CP and the care burden of their caregivers, exposing them to greater stress than before the lockdown.

The importance of BT therapy to treat these patients and the need to administer it regularly and without any important interruption are essential to not lose its clinical benefits, so any kind of treatment suspension or delay must be absolutely avoided.

## Data Availability

All data generated or analyzed during this study are included in this published article.
